# The significance of the best puncture side bone cement/vertebral volume ratio to prevent paravertebral vein leakage of bone cement during vertebroplasty: a retrospective study

**DOI:** 10.1186/s12891-023-06580-x

**Published:** 2023-06-09

**Authors:** Tao Gao, Zhi-Yu Chen, Tao Li, Xu Lin, Hai-Gang Hu, Xiang-Yu Wang, Jun Zeng, Chao Wu

**Affiliations:** 1 Orthopaedics of Zigong Fourth People’s Hospital, Zigong, 643000 Sichuan China; 2 Respiratory Medicine of Zigong Fourth People’s Hospital, Zigong, 643000 Sichuan China

**Keywords:** Vertebroplasty, Cement leakage in paravertebral veins, Volume of puncture-side bone cement, Puncture-side bone cement/vertebral volume ratio, Three-dimensional reconstruction

## Abstract

**Objectives:**

To verify the clinical significance of the best puncture-side bone cement/vertebral volume ratio (PSBCV/VV%) and bone cement leakage in paravertebral veins during vertebroplasty.

**Methods:**

This was a retrospective analysis of a total of 210 patients from September 2021 to December 2022, who were divided into an observation group (110 patients) and a control group (100 patients). In the observation group, patients’ preoperative computed tomography (CT) data were imported into Mimics software, and the VV was calculated using the three-dimensional (3D) reconstruction function. Then, based on the best PSBCV/VV% of 13.68% determined in a previous study, the optimal PSBCV to be injected during vertebroplasty was calculated. In the control group, vertebroplasty was performed directly using the conventional method. The incidence of cement leakage into paravertebral veins was observed postoperatively in both groups.

**Results:**

There were no statistically significant differences (P > 0.05) in the evaluated indicators between the two groups pre- or postoperatively, including the anterior vertebral margin height, mid-vertebral height, injured vertebral Cobb angle, visual analogue scale (VAS) score, and Oswestry Disability Index (ODI). Intragroup comparisons showed improvements in the anterior vertebral height, mid-vertebral height, injured vertebral Cobb angle, VAS score, and ODI after surgery compared with before surgery (P < 0.05). In the observation group, there were 3 cases of cement leakage into the paravertebral veins, for a leakage rate of 2.7%. In the control group, there were 11 cases of cement leakage into the paravertebral veins, for a leakage rate of 11%. The difference in the leakage rate between the two groups was statistically significant (P = 0.016).

**Conclusion:**

In vertebroplasty, preoperative VV calculations using Mimics software, combined with calculation of the PSBCV according to the best PSBCV/VV% (13.68%), can effectively prevent leakage of bone cement into paravertebral veins and further prevent serious life-threatening complications such as pulmonary embolism.

## Introduction

Vertebroplasty is a routine treatment for osteoporotic vertebral compression fractures (OVCFs), with advantages such as minimal trauma, rapid pain relief, and the ability to strengthen the vertebral body and re-establish spinal stability [1–3]. However, the problem of intraoperative cement leakage is still unavoidable. Bone cement leakage is defined as the presence of bone cement outside the cortical bone boundary of the operated segment or into the surrounding vessels on postoperative X-ray or CT sagittal, coronal and axial views. And when bone cement leakage in paravertebral veins can have serious and even life-threatening consequences [4–8]. Therefore, it is important to prevent the leakage of bone cement into paravertebral veins. Previous studies have suggested that the bone cement volume (BCV) is an independent risk factor for paravertebral leakage [9–12], but the volume of the injured vertebrae varies depending on sex, ethnicity, vertebral site, and degree of vertebral compression, making it difficult to determine a constant standard for the BCV. While recent studies have identified the BCV/VV% as an independent risk factor for paravertebral leakage [13–16], it has been found clinically that cement does not disperse uniformly in the vertebral body and instead tends to be concentrated on the puncture side. Furthermore, it has been found that bone cement leakage in paravertebral veins tends to occur on the puncture side, but there have been no relevant reports on the correlation between PSBCV/VV% and cement leakage in paravertebral veins. Previous studies by our team have shown that PSBCV/VV% is a high-risk factor for cement leakage in paravertebral veins during vertebroplasty and that the best PSBCV/VV% is 13.68%, with a significantly higher risk of paravertebral leakage when the PSBCV/VV% exceeds this value [17]. This study was conducted to further validate our previous findings. Mimics software was used to calculate the VV before the operation. Then, based on the best PSBCV/VV% of 13.68% determined in the previous study, we calculated the optimal amount of bone cement to be injected on the puncture side during vertebroplasty to further verify the clinical significance of the best PSBCV/VV% regarding the leakage of bone cement into paravertebral veins, thus providing guidance for the prevention of cement leakage into paravertebral veins and avoiding serious life-threatening complications such as pulmonary embolism in patients.

## Materials and methods

### General information

This was a retrospective analysis of patients treated from September 2021 to December 2022, who were divided into an observation group (110 patients) and a control group (100 patients). All patients had preoperative CT, magnetic resonance imaging (MRI), and bone density examination findings confirming OVCFs. A total of 210 patients were enrolled, including 69 males and 141 females, aged 55–92 years, with a mean age of 72.9 years. All patients were treated with percutaneous vertebroplasty (PVP) surgery. In the observation group, patients’ preoperative CT data were imported into Mimics software, and the VV was calculated using the 3D reconstruction function. Then, based on the best PSBCV/VV% of 13.68% determined in the previous study, the optimal amount of PSBCV during vertebroplasty was calculated. In the control group, vertebroplasty was performed directly using the conventional method. The study was approved by the ethics committee of our hospital.

### Inclusion and exclusion criteria

The inclusion criteria were as follows: (1) clear history of trauma, including low-energy injuries; (2) low back pain with localized pressure pain in the spinous process; (3) MRI scans showing a low signal on T1-weighted images and a high signal on T2-weighted images; (4) bone density results suggesting osteoporosis; (5) no signs of spinal cord or nerve damage.

The exclusion criteria were as follows: (1) patients younger than 55 years; (2) a severe fracture causing puncture difficulty; (3) infection at the puncture site or uncontrolled infection in other systemic systems; (4) severe cardiopulmonary comorbidity or serious coagulopathy; (5) pathological fractures due to spinal tumours.

### Surgical interventions

All surgeries were performed by experienced physicians. Bone cement and surgical instruments were supplied by the same company (Via Andrea Doria). Each patient was placed in a prone position with a pillow under the thorax and anterior superior iliac spine to bring the spine into a hyperextended position and complete the postural repositioning. A unilateral approach for arch root puncture was applied in all patients. C-arm fluoroscopy was used to locate the skin penetration point 1 cm lateral to the pedicle projection, and the patient was anaesthetized layer by layer with 1% lidocaine. Under C-arm fluoroscopy, puncture into the pedicle through the outer edge of the pedicle at 10 o’clock or 2 o’clock was performed. The needle was adjusted so that the tip of the needle was positioned at the inner edge of the arch on frontal fluoroscopy and at the posterior wall of the vertebral body on lateral fluoroscopy, and the needle was advanced to puncture the anterior 1/3 of the vertebral body. Then, the injection working channel was established, and polymethyl methacrylate (PMMA) bone cement was injected until the bone cement was close to the posterior wall of the vertebral body. In the observation group, preoperative PSBCV was calculated based on VV and the optimal PSBCV/VV% (13.68%), intraoperative cement injection volume was recorded, anteroposterior X-ray fluoroscopy was required while injecting the bone cement, and the proportion of intraoperative PSBCV to the injected cement volume was initially evaluated based on the cement dispersion, so that intraoperative PSBCV was judged and controlled the actual PSBCV did not exceed the PSBCV calculated preoperatively. If frontal fluoroscopy reveals that the bone cement is predominantly distributed on the punctured side, an angled guide pin can be used to establish contralateral access [18], and then cement injection can continue. In the control group, there was no need for anteroposterior fluoroscopy during the injection of bone cement, and there was no need to deliberately control the amount of bone cement injected on the puncture side. After the bone cement was completely solidified, the working sleeve was rotated and pulled out to prevent bone cement deformation and wrapped with sterile auxiliary materials after disinfection.

### Outcome measurements

**Fig. 1:**
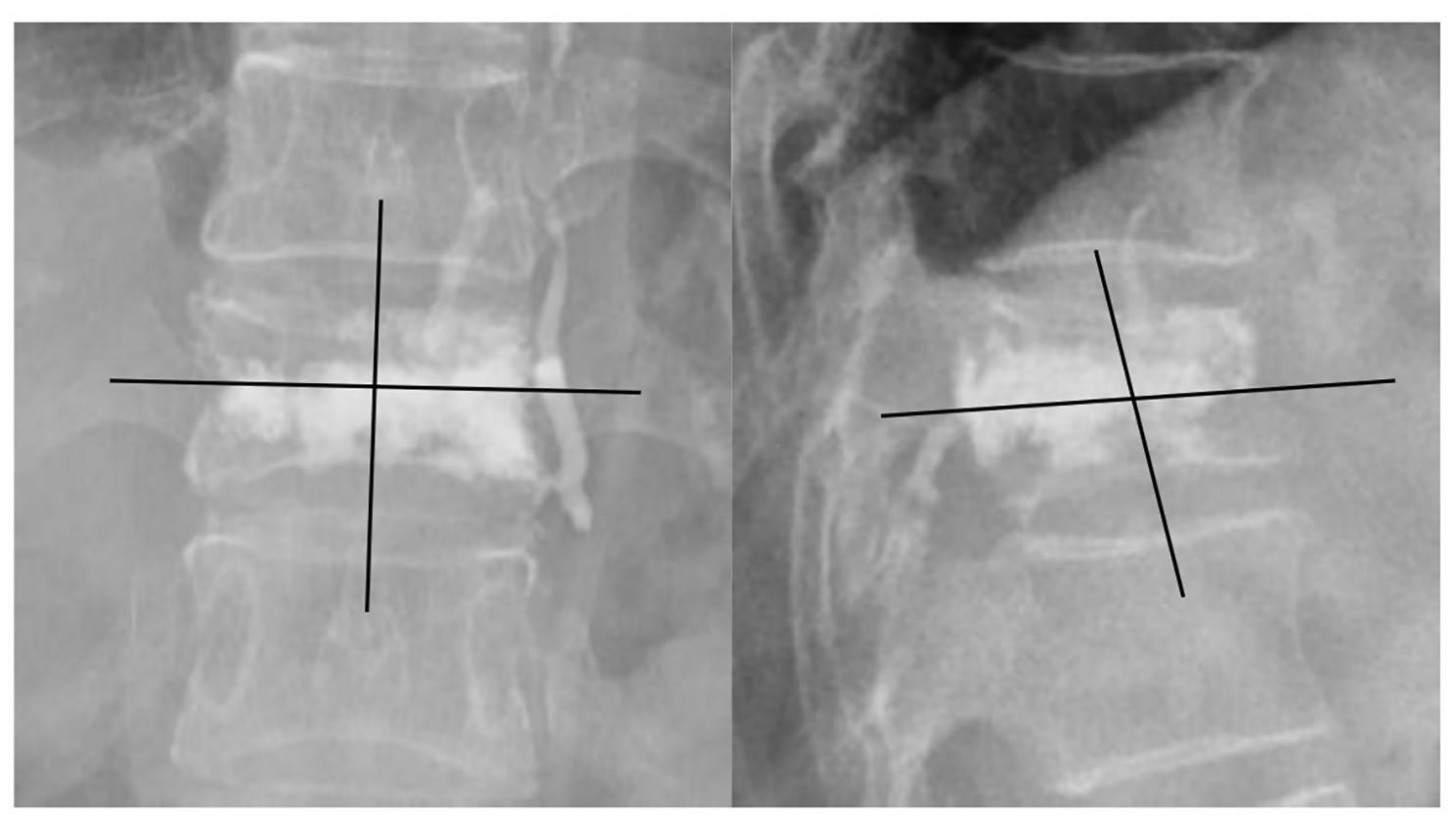
The vertebra was divided into 4 quadrants on lateral and anteroposterior radiographs. Each quadrant filled more than 1/3 with bone cement was scored as 1 point, and the total score was the bone cement distribution score

The general clinical data of patients in the two groups were recorded, including sex, age, body mass index (BMI), bone mineral density, surgical segment, operation time, and preoperative and postoperative vertebral anterior height, mid-vertebral body height, posterior injured vertebral Cobb angle, VAS score, and ODI. According to the results of postoperative anteroposterior and lateral X-rays, the dispersion of vertebral bone cement was recorded via the 8-point method [19] (Fig. 1).

Two physicians imported the preoperative CT data into Mimics 21.0 software and calculated the VV using the 3D reconstruction function. Then, based on the best PSBCV/VV% of 13.68% determined in the previous study, the optimal amount of bone cement to be injected on the puncture side during vertebroplasty was calculated. After the operation, the CT data of patients in the two groups were imported into Mimics software to calculate the BCV and the PSBCV, and the averages of the values determined by the two physicians were taken as the recorded results. Whether paravertebral venous leakage had occurred was determined according to the postoperative CT results and recorded. The CT system used was a SIEMENS SOMATOM Force scanner.

**Fig. 2:**
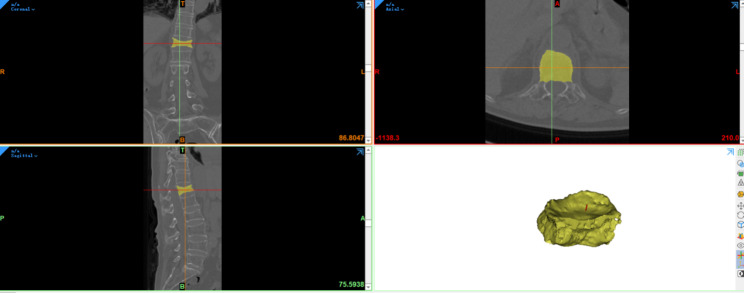
Calculation of the VV using Mimics software

**Fig. 3:**
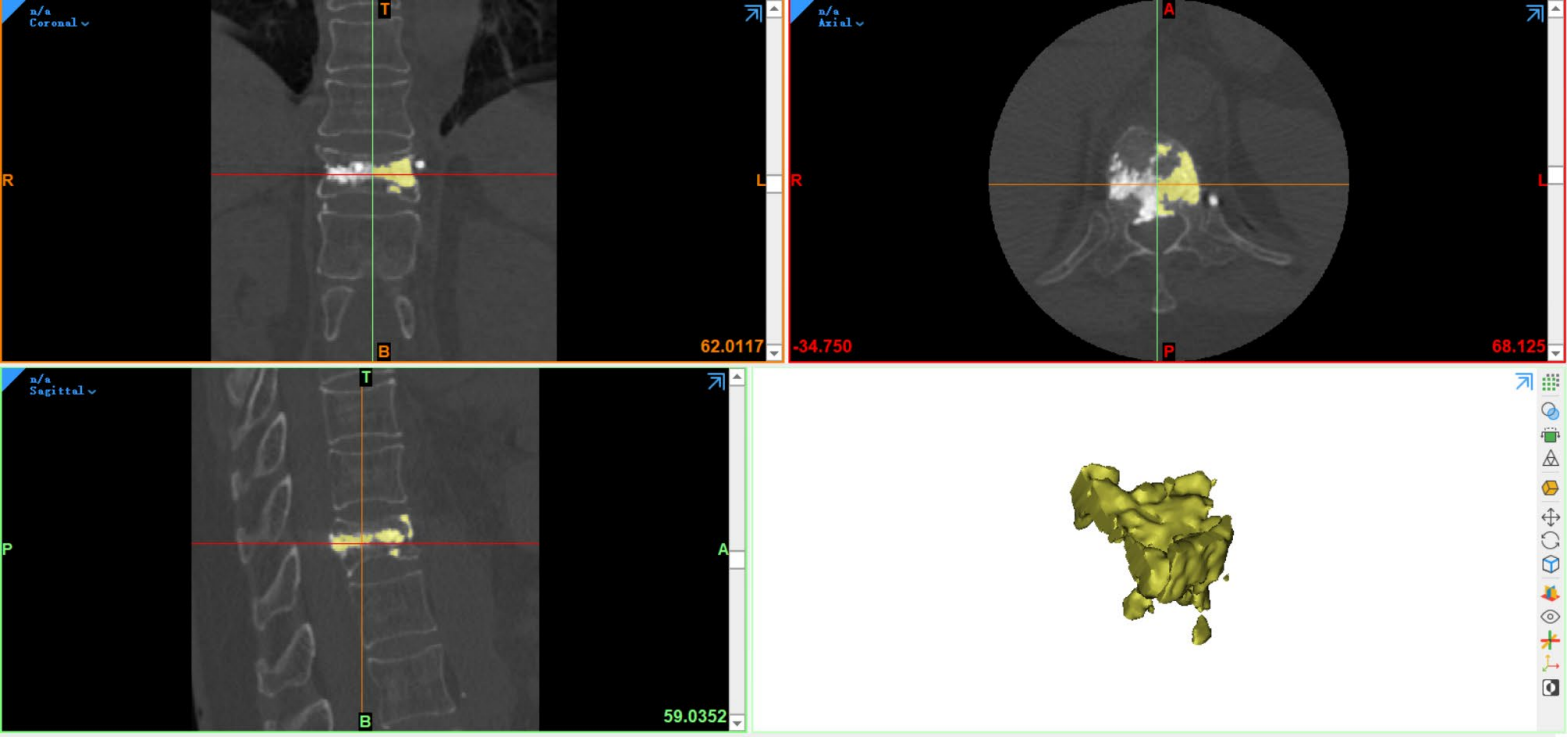
Calculation of the PSBCV using Mimics software

Patients’ preoperative CT data were imported into Mimics software, which was used to calculate the VV (Fig. 2), as described in our previous study [17]. Using a calculator, the optimal PSBCV was calculated before the operation based on the best PSBCV/VV% of 13.68%. After the operation, the postoperative CT data of the patients were imported into Mimics software, which was used to calculate the PSBCV (Fig. 3), as described in our previous study [17]. The midpoint of the upper and lower vertebral endplates was marked on the coronal CT scan, and the vertebrae were divided into left and right parts; the PSBCV was defined as the volume of cement in the half of the vertebral body on the side of the access point (i.e., the puncture side) [17]. Two resident physicians jointly observed postoperative anteroposterior and lateral X-rays and CT 3D reconstructions and screened out patients with paravertebral venous leakage, and the paravertebral venous incloud the anterior external venous plexus, basivertebral vein, anterior internal venous plexus, segmentalvein, inferior caval vein, or (hemi)azygos vein.

### Statistical analysis

The normality of data distributions was detected by the K-S test. All descriptive follow-up data are expressed as means ± SDs. All differences in continuous variable between the two groups were assessed by paired t tests. Differences in sex and the leakage rate between the two groups were determined by x^2^ test. P values were two-sided, and a P value < 0.05 was considered to indicate a significant difference. SPSS (version 24.0) software was used for all analyses. Representations of the data were drawn using GraphPad Prism 9 software.

## Results

From September 2021 to December 2022, a total of 210 patients with OVCFs were enrolled, including 110 patients in the observation group and 100 patients in the control group. The operation was completed normally in all patients.

### Baseline characteristic analysis

There was no significant difference in age, sex, BMI, bone mineral density, or operation time between the two groups (P > 0.05, Table 1).

**Table 1:** Patients’ basic characteristics

Characteristics	Observation group	Control group	*t/χ2*	*P*
Age (years)	73.04 ± 8.33	72.81 ± 8.16	0.199	0.843
Sex (M/F)	36/74	33/67	0.002	0.966
BMI (kg/m^2^)	22.17 ± 3.67	22.94 ± 3.74	-1.503	0.134
Bone density	-3.16 ± 0.56	-3.19 ± 0.52	0.316	0.753
Operating time (min)	31.63 ± 5.60	30.15 ± 6.50	1.768	0.078

### Imaging analysis

**Fig. 4:**
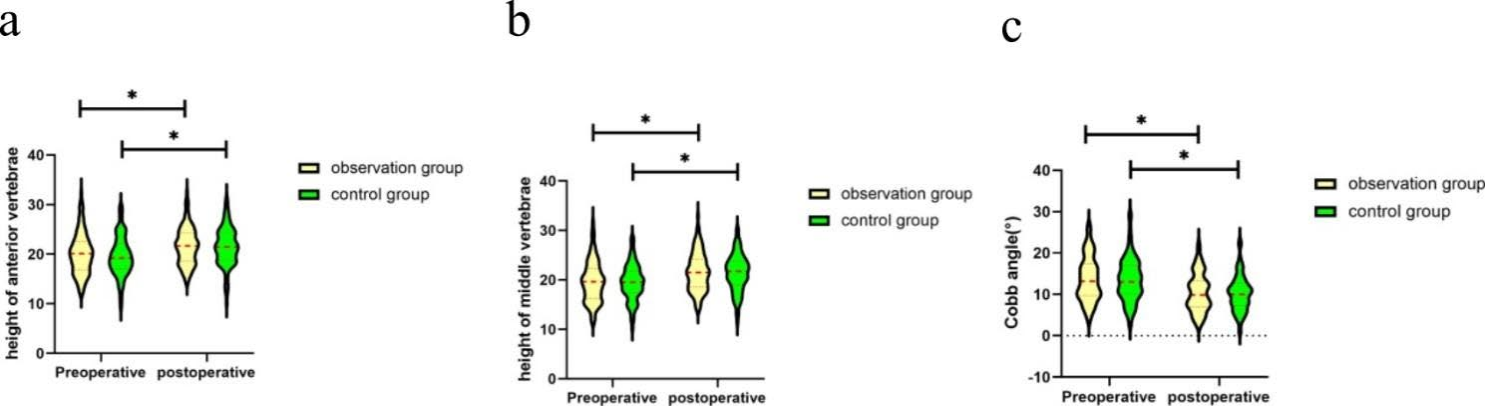
Pre- and postoperative anterior vertebral height (**a**), mid-vertebral height (**b**), and Cobb angle (°). *Indicates a difference within a group between before and after the operation (P < 0.001)

There was no significant difference in the height of the anterior edge of the vertebral body, the height of the middle part of the vertebral body, or the Cobb angle of the injured vertebra between the two groups before or after the operation (P > 0.05). Intragroup comparisons showed statistically significant improvements in the height of the anterior edge of the vertebral body, the height of the middle part of the vertebral body, and the Cobb angle of the injured vertebra after surgery compared with before surgery (P < 0.05, Table 2; Fig. 4).

**Table 2:** Comparison of imaging characteristics between the two groups

Characteristics	Observation group	Control group	*t*	*P*
Preoperative anterior vertebral height (mm)	20.11 ± 4.18	19.60 ± 3.97	0.909	0.364
Preoperative mid-vertebral height (mm)	19.65 ± 4.14	19.51 ± 3.64	0.262	0.794
Postoperative anterior vertebral height (mm)	21.66 ± 3.72	21.50 ± 3.89	0.312	0.755
Postoperative mid-vertebral height (mm)	21.61 ± 3.89	21.42 ± 3.73	0.362	0.718
Preoperative Cobb angle (°)	13.73 ± 5.38	13.54 ± 5.21	0.256	0.798
Postoperative Cobb angle (°)	10.48 ± 4.64	10.27 ± 4.32	0.333	0.740

### Comparison of prognosis

**Fig. 5:**
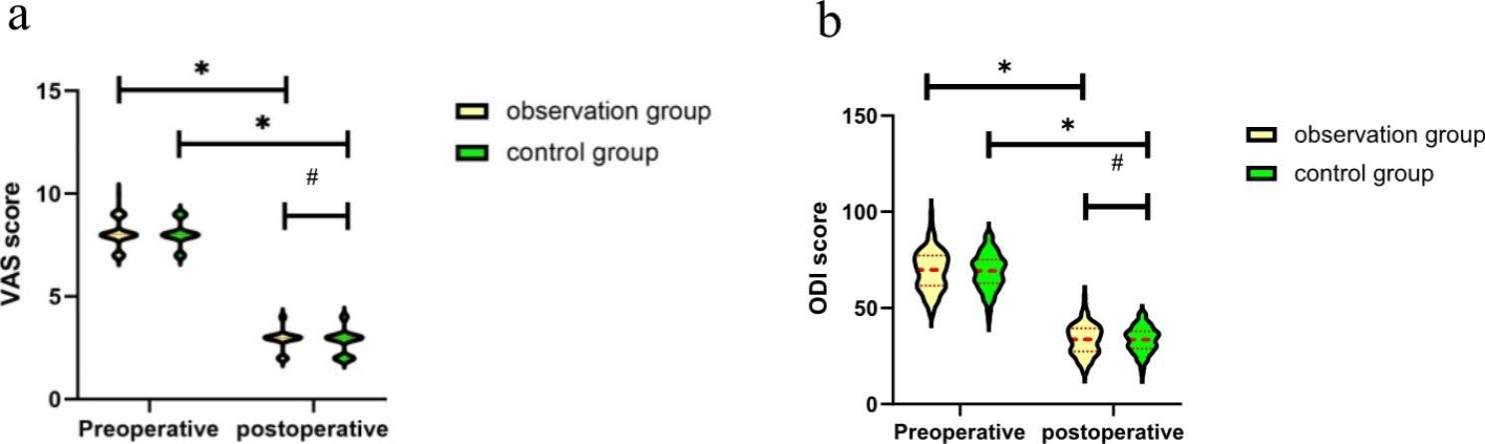
Pre- and postoperative VAS score (**a**) and ODI (**b**). *Indicates a difference within a group between pre- and postoperation (P < 0.001). ^**#**^Indicates no difference between the two groups postoperation

There was no significant difference in the VAS score or ODI between the two groups before or after surgery (P > 0.05). The postoperative VAS score and ODI were significantly improved compared with those before the operation within the groups (P < 0.05, Table 3; Fig. 5).

**Table 3:** Comparison of prognosis between the two groups

Characteristics	Observation group	Control group	*t*	*P*
Preoperative VAS score	8.06 ± 0.65	8.05 ± 0.61	0.156	0.876
Postoperative VAS score	2.90 ± 0.52	2.78 ± 0.61	1.518	0.131
Preoperative ODI	69.44 ± 10.46	69.07 ± 9.32	0.272	0.786
Postoperative ODI	33.43 ± 7.91	33.44 ± 6.74	-0.008	0.994

### Comparison of BCV and paravertebral leakage rate

There was no significant difference in the preoperative VV or BCV between the two groups. The bone cement distribution score in the observation group was higher than that in the control group, while the PSBCV was smaller in the observation group than that in the control group, and both differences were statistically significant (P < 0.05). In the observation group, there were 3 cases of bone cement leakage into paravertebral veins, for a leakage rate of 2.7%. In the control group, there were 11 cases of bone cement leakage into paravertebral veins, for a leakage rate of 11%. The difference in the rate of bone cement leakage into paravertebral veins between the two groups was statistically significant (P < 0.05, Table 4). A total of 14 patients in both groups were found to have paravertebral vein leakage. All patients had no symptoms of pulmonary embolism, such as coughing, coughing up blood, chest tightness, shortness of breath and rapid heart rate. Therefore no post-operative chest CT examination was performed.

**Table 4:** Comparison of BCV and paravertebral leakage rate between the two groups

Characteristics	Observation group	Control group	*t/χ2*	*P*
Bone cement distribution score	7.53 ± 0.66	7.02 ± 0.89	4.729	0.000
VV (cm^3^)	24.81 ± 2.58	24.70 ± 2.42	0.304	0.761
PSBCV calculated preoperatively (cm^3^)	3.39 ± 0.35	3.38 ± 0.33	0.304	0.761
BCV (cm^3^)	4.97 ± 1.18	5.03 ± 1.19	-0.368	0.713
Actual PSBCV (cm^3^)	2.95 ± 0.51	3.66 ± 0.54	-9.820	0.000
Paravertebral leakage	3	11	5.761	0.016

## Discussion

### Pathological analysis of bone cement leakage into paravertebral veins

The internal and external vertebral veins, vertebral body veins, and their connecting veins together constitute the venous system of the vertebral body. The vertebral body veins are radially shaped, with veins on the anterolateral side communicating with prevertebral veins through small venous channels and posterior veins communicating with branches posterior to the ligamentum flavum [20]. The veins of the vertebral body have no venous valve structures and allow flow in both directions. Therefore, when bone cement enters the vertebral body venous system, it can easily be transferred to other locations, enter the azygos vein, return to the right heart through the inferior vena cava, and finally enter the pulmonary blood vessels [7, 21]. In a study by Hsieh et al. [22], all 9 cases of pulmonary embolism after vertebroplasty were caused by paraspinal leakage. Even in patients with atrial septal defects or ventricular septal defects, bone cement emboli may enter the systemic circulation, causing cardiac, renal, or cerebral embolism [8, 23, 24]. The reported rates of bone cement leakage-induced pulmonary embolism have varied, ranging from approximately 1-26.0% [25–29]. Pulmonary embolism caused by bone cement leakage usually causes no or only mild clinical symptoms, which is why most patients do not undergo chest imaging after vertebroplasty; thus, it is estimated that the actual incidence of pulmonary embolism due to bone cement leakage may be higher than reported rates [6, 30–32]. Luetmer et al [28] performed lung CT examinations on 244 patients who had undergone PVP surgery, and the results revealed bone cement emboli in the lungs of 23 (9.4%) patients. Bone cement fragments usually do not cause acute inflammatory reactions in lung tissue, which may be related to the good histocompatibility of bone cement materials [33, 34]. However, some patients will show pulmonary symptoms, such as cough, haemoptysis, chest tightness, shortness of breath, and rapid heart rate. When these symptoms appear after PVP, the possibility of bone cement pulmonary embolism should be considered and evaluated by further pulmonary imaging [35]. Some bone cement pulmonary embolisms can also cause haematological abnormalities, such as heart failure, pulmonary hypertension, and shock, or even respiratory and cardiac arrest [6–8]. Therefore, avoiding bone cement venous leakage is particularly important.

### Risk factors for cement leakage into paravertebral veins

There are many risk factors for bone cement leakage into paravertebral veins, such as the internal pressure of the vertebral body, the amount of bone cement injected, the BCV/VV%, the angle and location of working tube inside the vertebral body, the type of fracture, the cement spread, etc. Among them, the internal pressure of the vertebral body is an important factor. As the venous pressure in the vertebral body is lower than the arterial pressure, bone cement tends to leak into veins but not into arteries [36]. Aebli et al [37] performed vertebroplasty on sheep vertebral bodies and monitored the leakage of bone cement under different pressure states; the results showed that increased pressure in the vertebral body induced, bone cement leakage into epidural and paravertebral veins. The wall of veins in the vertebral body is thin, and when the intramedullary pressure exceeds a certain range during the injection of bone cement, small grains of bone cement will be forced into the veins, resulting in cement leakage into paravertebral veins. With higher pressure in the vertebral body, the BCV that can be accommodated in the same space decreases, and the same BCV requires greater pressure to inject, which increases the risk of bone cement leakage into paravertebral veins. In PVP, the pressure in the vertebral body, and thus the risk of bone cement leakage into paravertebral veins, can be reduced by repeatedly injecting a small amount of cement slowly [38]. Second, the amount of bone cement injected is also considered to be a risk factor for the leakage of bone cement into paraspinal veins. Several studies have shown that the greater the BCV injected, the higher the risk of bone cement leakage into paravertebral veins [9–12]. Zhu et al. [11] found that to avoid bone cement leakage, less than 3.5 ml of bone cement is safe in the thoracic spine and less than 4 ml is safe in the lumbar spine. Tang et al. [10] showed that the BCV injected was an independent risk factor for venous leakage. When the BCV increased by 1.0 ml, the risk of venous leakage increased by 8%. A study by Fu Z et al. [12] also showed that the BCV injected was closely related to the incidence of cement leakage into paravertebral veins. Unfortunately, this study did not give a recommended value for the BCV. Because the vertebral body volume of each patient is greatly affected by age, sex, race, fracture segment, etc., it is unscientific to simply consider the impact of the BCV on the rate of bone cement leakage; however, the BCV/VV% is not affected by the abovementioned factors. Therefore, in recent years, the effect of the BCV/VV% on bone cement leakage has gradually become a hot topic. Barriga-Martín et al. [14] analysed the volume of 41 vertebral bodies undergoing PVP surgery and found that the average vertebral body volume was 26.1 ml, the average BCV injected was 2.0 ml, and the average BCV/VV% was 9%; in that study, bone cement leakage into the vascular system occurred in a total of 19.5% of the patients. Jin et al. [39] found that the risk of pulmonary embolism was significantly increased when the BCV/VV% exceeded 21% at the T11-L1 level and suggested that the best BCV/VV% to relieve pain and prevent leakage-related complications was 11.65%. Nieuwenhuijse et al. [13] suggested that the best BCV/VV% in vertebroplasty is 24%, and a study by Kwon et al. [15] indicated that the optimal BCV/VV% in vertebroplasty is 27.8%, with a sensitivity of 80% and a specificity of 87.5%. However, the BCV in the above studies was calculated by the amount of bone cement injected during the operation, while the VV was calculated by a mathematical model. Notably, due to bone cement leakage, bone cement residue may be present in the puncture sleeve, and the BCV may not be equal to the injected BCV. Additionally, the VV calculated by the mathematical model may not be exactly equal to the actualVV; thus, BCV/VV% calculated in this way may not be accurate. In previous research, our team imported the postoperative CT data of patients into Mimics software, through which the actual BCV in the vertebral body and the real VV could be accurately calculated and thus the BCV/VV% could be accurately calculated. During vertebroplasty, the bone cement is often not evenly distributed in the vertebral body, and usually more bone cement is distributed on the puncture side than on the contralateral side; thus, bone cement leakage into paraspinal veins usually occurs on the puncture side. Our team’s previous research showed that the PSBCV has a greater effect on paravertebral venous leakage than the BCV [17].

### Application of the best PSBCV/VV% in vertebroplasty

Our team’s previous research showed that the PSBCV/VV% is a high-risk factor for bone cement leakage into paravertebral veins in vertebroplasty and that the best PSBCV/VV% is 13.68%. When the PSBCV/VV% exceeds this value, the risk of cement leakage into paravertebral veins increases significantly [17]. This study further validated this research result. In this study, the cement distribution score was significantly better in the observation group than in the control group, and the rate of paravertebral leakage was also significantly lower in the observation group than in the control group, suggesting that intraoperative control of the PSBCV/VV% to less than 13.68% can significantly reduce paravertebral leakage of bone cement. Therefore, it is recommended to use Mimics software to calculate the VV of the injured vertebra before the operation and use the best PSBCV/VV% of 13.68% to calculate the PSBCV. Anteroposterior X-ray radiography was applied during the operation to estimate the PSBCV according to the distribution of bone cement, and the PSBCV was controlled to not exceed the volume calculated before the operation. Especially when the anteroposterior X-ray view shows that the bone cement distribution is concentrated on the puncture side, a curved-angle diffusion device can be used to establish a bone cement tunnel leading to the opposite side, which would allow the bone cement to diffuse to the opposite side and thereby reduce the BCV on the puncture side.

### Research shortcomings

One shortcoming of this study was that some intraoperative bone cement would flow completely elsewhere through the paravertebral vein and could not be detected on postoperative CT. Therefore, the incidence of actual paravertebral vein leakage of bone cement is likely to be higher. Another shortcoming of this study was that the PSBCV could only be estimated based on the diffusion of bone cement on the anteroposterior X-ray view and couldnot be calculated accurately. In the future, our team plans to use intraoperative 3D CT to accurately calculate the PSBCV to further improve the accuracy of the experimental method.

## Conclusion

Mimics software was used to calculate the VV before vertebroplasty, and the best PSBCV/VV% (13.68%) was used to calculate the PSBCV for injection. During the operation, the PSBCV was controlled to not exceed the volume calculated before the operation. This method can effectively prevent the leakage of bone cement into paravertebral veins, further helping to prevent life-threatening serious complications such as pulmonary embolism.

## Data Availability

All data generated or analysed during this study are included in this published article.

## List of abbreviations

3D: Three-dimensional
BCV: Bone cement volume
BMI: Body mass index
CT: Computed tomography
MRI: Magnetic resonance imaging
ODI: Oswestry dysfunction index
OVCF: Osteoporotic vertebral compression fractures
PSBCV/VV%: Puncture-side bone cement/vertebral volume ratio
PSBCV: Puncture-side bone cement volume
VAS: Visual analogue scale
VV: Vertebral volume
